# Purple Basil (*Ocimum basilicum* L.): A Source of Bioactive Molecules for Functional Foods and Dietary Strategies

**DOI:** 10.1155/ijfo/5879376

**Published:** 2026-07-22

**Authors:** Rosane Patricia Ferreira Chaves, Elivaldo Nunes Modesto Junior, Gustavo Araujo Pereira, Rosinelson da Silva Pena

**Affiliations:** ^1^ Graduate Program of Food Science and Technology (PPGCTA), Institute of Technology (ITEC), Federal University of Pará (UFPA), Belém, Pará, Brazil, ufpa.br; ^2^ Faculty of Food Engineering (FEA), Institute of Technology (ITEC), Federal University of Pará (UFPA), Belém, Pará, Brazil, ufpa.br

**Keywords:** aromatic herbs, dried herbs, essential oil, leaves, minimally processed

## Abstract

This review addresses the nutrients, bioactive compounds, and the potential use in the food industry of purple basil (*Ocimum basilicum* L.). Purple basil is recognized for its high content of bioactive compounds, notably phenolic compounds, chlorophylls, and carotenoids, which contribute to its high antioxidant and antimicrobial properties. Studies have explored the use of purple basil in various forms, including dried leaves, essential oil, infusions, and minimally processed products, demonstrating its versatility in food formulations. Research highlights that incorporating purple basil into products such as ice cream, cheese, yogurt, and kombucha enhances their nutritional and functional properties by increasing antioxidant activity and phenolic compound content. Additionally, its application in meat preservation has shown effectiveness in inhibiting microbial growth and delaying lipid oxidation, whereas its inclusion in biodegradable films and active food packaging offers innovative solutions for food quality monitoring and shelf life extension. Purple basil emerges as a promising natural ingredient for the food industry and gastronomy due to its chemical profile and health benefits—supporting the development of healthier, functional, and sustainable food products. Further studies are encouraged to optimize processing techniques and fully harness the potential of *O. basilicum* in diverse food application.

## 1. Introduction

Aromatic plants and their derivative products, such as essential oils, extracts, and infusions, are gaining prominence in the international market due to their applications in traditional medicine, food preservation, and cosmetic formulations [1, 2]. These plants are rich in bioactive compounds, primarily phenolics (both volatile and nonvolatile), whose consumption has been linked to health benefits, including antioxidant properties, anti‐inflammatory activity, and the prevention of cardiovascular diseases [3, 4].

Purple basil (*Ocimum basilicum* L.), a cultivar of common basil, is an aromatic herb widely used as a seasoning in cooking, in tea preparations, and for essential oil production [5, 6]. It is referred to as “purple” because it produces both purple and white flowers [7, 8]. It has significant potential in the food and pharmaceutical industries due to its diverse bioactive compounds, including phenolic acids, flavonoids, tannins, polyphenols, terpenes, alkaloids, and saponins [5, 9, 10], making it a promising functional ingredient.

The chemical composition of purple basil varies depending on factors such as cultivar, geographical origin, cultivation conditions, and harvest time [11, 12]. Research has demonstrated its antioxidant [13, 14], anti‐inflammatory [15, 16], and antimicrobial [17, 18] activities.

Several scientific studies have explored the potential applications of basil in the food industry due to its biological properties and chemical composition. Ebrahimi et al. [19] developed a pH‐sensitive film packaging made with ethanolic extract of purple basil and bovine gelatin, designed to monitor the freshness of chicken fillets during storage. The incorporation of purple basil helped regulate microbial growth and antioxidant activity, with anthocyanins serving as indicators of deterioration through a color change from purple to green. Similarly, Hamad et al. [20] investigated the use of *Ocimum africanum* Lour. for chicken meat preservation due to its antimicrobial properties. Popescu et al. [21] microencapsulated basil extract and incorporated it into cream cheese at varying concentrations, observing that the microcapsules inhibited postfermentation, improved texture and water retention, and extended the product′s shelf life.

In this context, this review aims to provide a comprehensive scientific analysis of *O. basilicum*, focusing on its chemical composition and potential applications in the food industry. By compiling and evaluating recent studies, this work highlights the functional benefits of purple basil and its growing relevance as a natural ingredient in food formulations.

## 2. Purple Basil (*O. basilicum* L.)

The Lamiaceae family includes a wide cultivar of plants known for their medicinal and culinary uses, such as mint (*M* *e* *n* *t* *h* *a* × *v* *i* *l* *l* *o* *s* *a* Huds), lemon balm (*Melissa officinalis* L.), boldo (*Plectranthus barbatus* Andrews), and basil (*O. basilicum* L.) [22, 23].

The genus *Ocimum* comprises approximately 150 species, with five being the most well‐known: *O. basilicum*, *O. gratissimum*, *O. americanum*, *O. sanctum*, and *O. kilimandscharicum* [24, 25]. This genus exhibits significant morphological variation, including differences in leaf arrangement, inflorescence structure, flower and fruit morphology, and variations in chemical composition. Additionally, *Ocimum* species display substantial intraspecific diversity due to their high cross‐pollination capacity, which enhances the phenotypic differences among plants within this genus [24–26].

**Figure 1:**
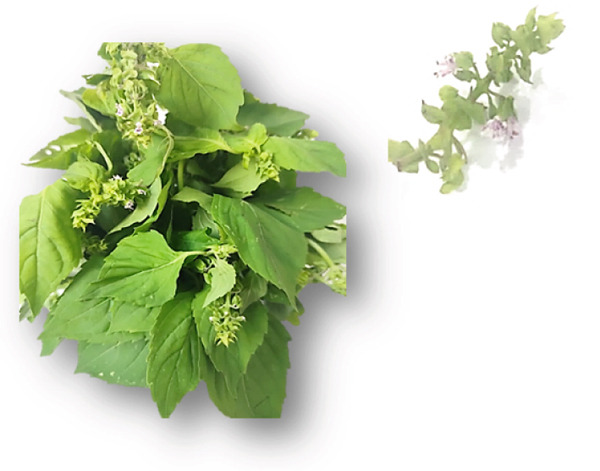
Purple basil (*O. basilicum* L.). Registration code MFS009423, Marlene Freitas da Silva (MFS) herbarium of the State University of Pará (UEPA).

Among the various *Ocimum* species, *O. basilicum* is the most commercially cultivated and is further classified into multiple cultivars based on leaf color, size, stem characteristics, and flower morphology. Purple basil comprises several varieties distinguished by green or purple leaf coloration and by flower color. According to Darrah′s classification, Carović‐Stanko et al. [7] reported six morphologies, including purple basil. Purple basil cultivated in the Amazon region, despite having green leaves, is regionally known as purple basil because of its purple and white flowers [7, 8] (Figure 1). This cultivar is an annual, erect aromatic herb that can grow up to 90 cm in height. The plant has oval‐lanceolate green leaves with serrated margins and a glossy, fragrant surface, and its flowers are arranged in simple clusters displaying both white and purple hues [5, 8].

Purple basil is also known as common basil. It is widely distributed across tropical and subtropical regions, commonly found in Africa, Asia, South America, and the Mediterranean [5, 6, 27]. This species is rich in secondary metabolites, primarily polyphenols, including phenolic acids, flavonoids, and anthocyanins. In traditional medicine, it has been used as a stimulant, as well as for the treatment of hypertension and renal failure. It is also employed for cough relief and in therapeutic baths to alleviate colds [28, 29]. Additionally, purple basil is widely used as a culinary ingredient and valued for its essential oils, which make it highly sought after in the perfumery, cosmetics, pharmaceutical, and food industries [28, 30].

Products containing purple basil have gained increasing attention due to their bioactive properties. Stanojević et al. [31] reported that the essential oil extracted from *O. basilicum* leaves contains 65 phytochemicals, with linalool (31.6%) and methyl chavicol (23.8%) being the major components, contributing to its antioxidant and antimicrobial properties. Bajomo et al. [8] analyzed the chemical composition of *O. basilicum* leaves and found significant concentrations of caftaric, caffeic, and ferulic acids. Similarly, Genc et al. [32] identified rosmarinic acid, rutin, 4‐hydroxybenzoic acid, caftaric acid, caffeic acid, gallic acid, and salicylic acid in purple basil leaves, further supporting its antioxidant potential.

In this context, purple basil shows significant potential for various industrial applications due to its chemical composition and bioactive properties. This review addresses the composition and functional uses of purple basil in food formulations aiming to elucidate its use in the food industry and gastronomy.

## 3. Chemical Composition of Purple Basil

Plants possess a highly diverse metabolic network, comprising both primary and secondary metabolism. Primary metabolites are essential compounds produced by all plant species, including carbohydrates, lipids, proteins, and nucleic acids, which are directly involved in vital physiological processes. In contrast, secondary metabolites are structurally complex, generally low‐molecular‐weight compounds with biological activity, playing a crucial role in ecological interactions and plant adaptation [33, 34].

Foods such as fruits, vegetables, cereals, and plant‐derived products are rich sources of secondary metabolites, classifying them as functional foods with bioactive properties. Upon ingestion, these compounds exert biological effects on human health, particularly as antioxidants, which are associated with the reduction of chronic noncommunicable diseases, including obesity, diabetes, cancer, and cardiovascular disorders [35, 36].

Plant species containing secondary metabolites offer a wide range of applications in the food industry, serving as natural colorants, flavor enhancers, stimulants, aromatic agents, preservatives, and antioxidants. Their incorporation into food products can extend shelf life by inhibiting lipid oxidation, thereby improving product stability and quality [37–40].

In this context, purple basil emerges as a promising aromatic herb for the food industry as it is rich in bioactive compounds, offering potential benefits for both human health and food preservation.

### 3.1. Phenolic Compounds

**Figure 2:**
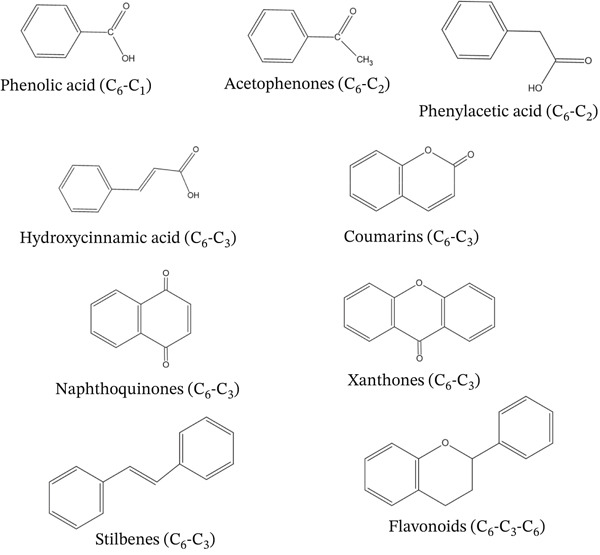
Chemical structure of the main groups of phenolic compounds.

Phenolic compounds are bioactive substances derived from plant metabolism, characterized by an aromatic ring with one or more hydroxyl groups, along with various functional substituents. These compounds exhibit diverse and multifunctional structures, ranging from simple monomeric molecules to highly polymerized forms. Their classification is primarily based on the number of carbon atoms in their structure (Figure 2) [41, 42].

The biosynthesis of phenolic compounds occurs via the shikimic acid pathway, in which a phosphoenolpyruvate (PEP) molecule from glycolysis binds to an erythrose‐4‐phosphate (E4P) molecule, derived from the pentose phosphate pathway, forming a seven‐carbon sugar known as 3‐deoxy‐D‐arabino‐heptulosonate‐7‐phosphate (DAHP). DAHP undergoes a series of enzymatic reactions catalyzed by 3‐dehydroquinate synthase, dehydroquinate dehydratase, and shikimate dehydrogenase, with the involvement of the reduced enzyme cofactor NADPH, leading to the formation of shikimate. From shikimate, three additional reactions occur, involving the enzymes shikimate kinase, 5‐enolpyruvylshikimate 3‐phosphate synthase, and chorismate synthase, along with ATP as an enzyme cofactor, ultimately producing chorismate. This compound serves as a precursor for the synthesis of the three aromatic amino acids: phenylalanine, tyrosine, and tryptophan. The formation of phenolic compounds begins with the deamination of phenylalanine, catalyzed by the enzyme phenylalanine ammonia‐lyase (PAL), leading to the production of cinnamic acid, which serves as a direct precursor for the biosynthesis of coumarins, phenylpropanoids, flavonoids, and lignins [43–45].

Phenolic compounds play a crucial role in plant growth and reproduction, acting as protective agents against ultraviolet radiation and plant pathogens [46]. Studies have demonstrated that, due to their antioxidant properties, phenolic compounds can prevent free radical formation or slow down oxidative processes in both plants and living organisms by neutralizing reactive species and exerting reducing activity. Dietary sources of phenolic compounds contribute to the repair of oxidative stress‐induced damage in tissues, significantly reducing inflammation, body mass, and blood pressure, as consequence lowering the risk of cardiovascular diseases. Additionally, these compounds protect biomolecules such as DNA from oxidative damage, playing a role in cancer prevention [47, 48].

Phytochemical analyses of *O. basilicum* revealed that phenolic compounds are predominant in its leaves, particularly flavonoids, phenolic acids, and polyphenolic tannins [5]. Table 1 summarizes the main phenolic compounds identified in basil leaves. Recent studies attribute antioxidant, antimicrobial, anticancer, anti‐inflammatory, neuroprotective, analgesic, and antihyperglycemic properties to the phenolic compounds present in *O. basilicum* leaves [6, 52, 53].

**Table 1:** Main phenolic compounds reported in *O. basilicum* leaves.

Sample	Analytical method	Phenolic compound	Content	References
*O. basilicum* leaves (extraction in methanol 98%)	HPLC‐ UV‐VIS	Gallic acid	6.5 mg/100 g	Ullah et al. [49]
		Caffeic acid	7.9 mg/100 g	
		Ferulic acid	4.9 mg/100 g	
		Sinapic acid	4.6 mg/100 g	
		Quercetin	3.4 mg/100 g	
		Apigenin	2.3 mg/100 g	

Leaves of *O. basilicum* cultivar “Classic Italian” (etanol extraction)	HPLC‐DAD	Linalool	31.51 mg/g	Romano et al. [50]
		Eugenol	27.27 mg/g	
		Rosmarinic acid	8.08 mg/g	
		Bornyl Acetate	6.02 mg/g	
		Eucalyptol	2.28 mg/g	
		Caffeic acid	0.97 mg/g	
		Chicoric acid	0.75 mg/g	
		Ferulic acid	0.17 mg/g	

Leaves of *O. basilicum* cultivar “Classic Italian” (supercritical CO_2_ extraction)	HPLC‐DAD	Linalool	35.08 mg/g	Romano et al. [50]
		Eugenol	25.42 mg/g	
		Rosmarinic acid	5.79 mg/g	
		Caffeic acid	1.69 mg/g	
		Eucalyptol	1.64 mg/g	
		Bornyl Acetate	1.56 mg/g	
		Chicoric acid	0.49 mg/g	
		Ferulic acid	0.13 mg/g	

Leaves of *O. basilicum* species purple basil (extraction in methanol/dichloroethano)	LC‐TOF‐MS	Rosmarinic acid	2610.15 mg/kg	Genc et al. [32]
		Rutin	669.77 mg/kg	
		4‐hydroxybenzoic acid	216.59 mg/kg	
		Caftaric acid	153.79 mg/kg	
		Caffeic acid	140.85 mg/kg	
		Gallic acid	101.77 mg/kg	
		Salicylic acid	100.77 mg/kg	

*O. basilicum* leaves (extraction in ethanol 70%)	HPLC‐UV	Ferulic acid	437.58 mg/g	Antonescu et al. [51]
		Routine	50.18 mg/g	
		Caffeic acid	29.09 mg/g	
		Catechin	12.49 mg/g	
		Syringic acid	12.01 mg/g	
		Chlorogenic acid	8.94 mg/g	
		Cinnamic acid	5.26 mg/g	

Abbreviations: HPLC‐DAD, high‐performance liquid chromatography with diode‐array detection; HPLC‐UV, high‐performance liquid chromatography with ultraviolet detection; HPLC‐UV‐VIS, high‐performance liquid chromatography with ultraviolet‐visible detection; LC‐TOF‐MS, liquid chromatography with a time‐of‐flight analyzer coupled to mass spectrometry.

Phenolic compounds also contribute to the aroma and flavor of foods, directly influencing their quality and biological effects. Bajomo et al. [8] demonstrated the correlation between phenolic acids and the antioxidant properties of *O. basilicum* varieties. Additionally, Teofilovic et al. [54] highlighted that extraction conditions, such as time, solvent polarity, and leaf granulometry, significantly impact the yield and composition of phenolic compounds obtained from purple basil.

Research suggests that matrices rich in phenolic compounds represent a promising approach in the food industry, as these natural compounds exhibit multiple properties, including antioxidant, antimicrobial, and anti‐inflammatory activities. However, handling, processing, and storage conditions can influence the stability of these compounds in food. Several factors affect the composition of bioactive compounds in plant‐based foods, including enzymatic reactions, pH, heat exposure during blanching and pasteurization, light, and physical processes such as milling and peeling [55].

During ripening and postharvest processing, increased enzymatic activity can compromise plant cell integrity, altering the phenolic profile through deglycosylation or oxidation reactions [55]. Polyphenol oxidase (PPO) and peroxidase (POD) are key enzymes associated with the degradation of phenolic compounds, leading to undesirable changes such as softening, darkening, and off‐flavors, which signal product deterioration [56]. Chaves et al. [57] demonstrated that convective drying led to significant degradation of phenolic compounds in purple basil. Their study indicated that drying temperature of 40°C minimized phenolic losses, preserving the bioactive potential of the plant.

Further research is needed to optimize both the extraction and preservation of phenolic compounds from *O. basilicum*. Do et al. [14] developed an optimized extraction process based on antioxidant activity. They evaluated the effects of different solvents (methanol, ethanol, acetone, and water) on the yield of total phenolic compounds and applied a central composite design to assess the influence of methanol concentration, solvent‐to‐sample ratio, temperature, and extraction time. The optimal extraction conditions reported were methanol‐to‐sample ratio of 44.6 mL/g, methanol concentration of 39%, temperature of 90.7°C, and extraction time of 3.15 h. Under these conditions, the extract yielded 4.55 mg GAE/g of total phenolic content.

Compared with other aromatic plants such as mint, the content of total phenolic compounds differs considerably. Brown et al. [58] reported a maximum total phenolic content of 109.98 mg GAE/g in mint and identified its polyphenol profile—caffeoylquinic acid 3.3, salvianic acid, rosmarinic acid, luteolin, salvigenin, chrysoeriol, thymonin, and carnosol—although these compounds were not quantified individually. In contrast, Table 1 shows that phenolic acids are predominant in basil.

### 3.2. Chlorophylls

Chlorophylls are green pigments located in the thylakoid membranes of chloroplasts. In plants, they are crucial for photosynthesis, facilitating the conversion of solar energy into chemical energy for carbohydrate production. Chlorophylls play a vital role in light absorption, excitation energy transfer, and charge separation within the photosystems [59, 60].

**Figure 3:**
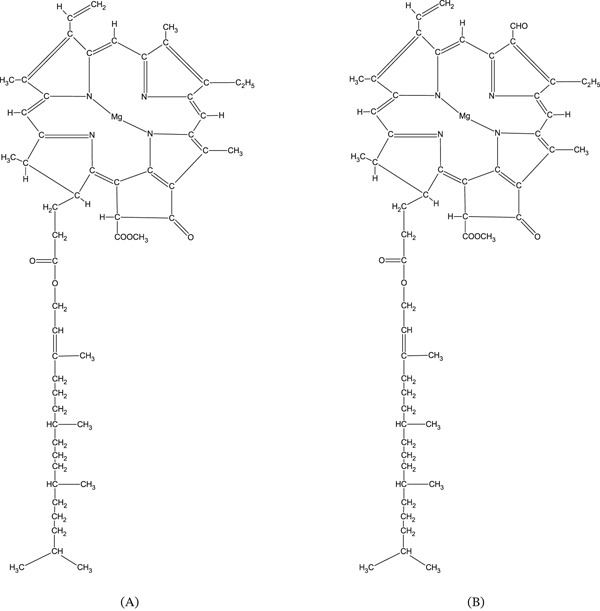
Chemical structure of (A) chlorophyll *a* and (B) chlorophyll *b*.

Structurally, chlorophylls are porphyrin molecules composed of four pyrrole rings connected by methylene bridges, four nitrogen atoms (N), and a central magnesium ion (Mg). Additionally, they contain a phytol group with a transconfigured double bond [59, 61]. Although more than 100 different chlorophyll structures exist in nature, chlorophyll *a* and chlorophyll *b* are the predominant forms found in plants (Figure 3) [62]. Chlorophyll *a* (Figure 3A) features a methyl group (CH_3_) as a functional group, giving it a blue‐green color, whereas chlorophyll *b* (Figure 3B) contains a formyl (CHO) group, which imparts a yellow‐green color [63].

As color is a significant attribute in the food industry, chlorophyll *a* and chlorophyll *b* have gained attention in research as potential natural dyes [64, 65]. They also serve as indicators of vegetable ripeness [62, 66]. Furthermore, studies have reported chlorophyll intake may help in the prevention of obesity [67, 68], act as a cancer prevention agent [68], and exhibit antimicrobial properties [62, 69].

Chlorophylls are sensitive to heat, light, acids, and enzymes, and their degradation can result in changes in color, texture, and nutritional value [70]. Heating plant tissue or adding acids can break down the chemical structure of chlorophylls, removing the Mg^2+^ ion or the ^−^CO_2_CH_3_ group, leading to the formation of pheophytin or pyropheophytin, which show olive green color [70, 71]. Enzymes such as chlorophyllase and oxidative enzymes such as lipoxygenase, chlorophyll oxidase, and POD also contribute to changes in the chemical structure and color of chlorophylls. Chlorophyllase removes the phytol group from chlorophyll, resulting in a green compound called chlorophyllide. Heat or acid treatments further lead to the formation of pheophorbide [64, 72].

The enzyme POD, which is commonly associated with the oxidation of phenolic compounds and indole acetic acid, can also degrade chlorophylls. In the presence of phenolic compounds with hydroxyl groups and superoxide anions, POD forms 132‐hydroxy‐chlorophyll *a*, an oxidized form of chlorophyll *a*, which can easily undergo cleavage in the macrocyclic ring, forming colorless fluorescent and nonfluorescent products with low molecular weight [59, 73].

Preserving chlorophyll is a challenge in maintaining the quality of vegetables during postharvest storage. Torales et al. [74] studied chlorophyll degradation in minimally processed arugula (*Eruca sativa* Mill) leaves, packaged with and without modified atmosphere. The authors found that modified atmosphere packaging, with low O_2_ and high CO_2_ content, provided a protective effect against chlorophyll degradation and delayed leaf yellowing.

Chen and Roca [75] researched chlorophyll degradation in edible seaweeds, such as nori (*Porphyra umbilicales*), sea lettuce (*Ulva* sp.), and kombu (*Laminaria ochroleuca*). They observed that cooking in heated water or microwaving caused pheophytinization and decarboxymethylation in the algae. Their findings indicated that chlorophyll oxidation altered the nutritional and biological properties of the algae.

Few scientific studies have evaluated the extraction, concentration, and degradation of chlorophylls in *O. basilicum* leaves. Carvalho et al. [76] compared with chlorophyll content of fresh and freeze‐dried *O. basilicum* leaves and found that, although freeze‐drying is a low‐temperature process, it still led to chlorophyll degradation. Fresh leaves contained 2287.8 *μ*g/100 g of chlorophyll *a* and 2607.4 *μ*g/100 g of chlorophyll *b*; however, after freeze‐drying, the levels decreased to 1003.8 and 2287.8 *μ*g/100 g, respectively. These results indicate that chlorophyll *a* is more sensitive to freeze‐drying than chlorophyll *b*. Similarly, Sharma et al. [77] evaluated basil leaves subject to microwave drying, sun drying, and oven drying at 45°C, 50°C, and 55°C, and reported that all methods induced chemical changes, including degradation of chlorophyll *a* and chlorophyll *b*.

Color is an important sensorial aspect of food, especially for edible leaves. Purple basil can be processed (minimally processed or dried) to improve shelf life, yet chemical changes should be considered in processing to obtain a stable and functional product. As previously mentioned, basil processing promotes changes in its chemical composition, such as degradation of phenolic compounds and chlorophyll. Despite the decrease in its bioactive content, processed purple basil contains a remarkable content of functional compounds [78].

### 3.3. Carotenoids

**Figure 4:**
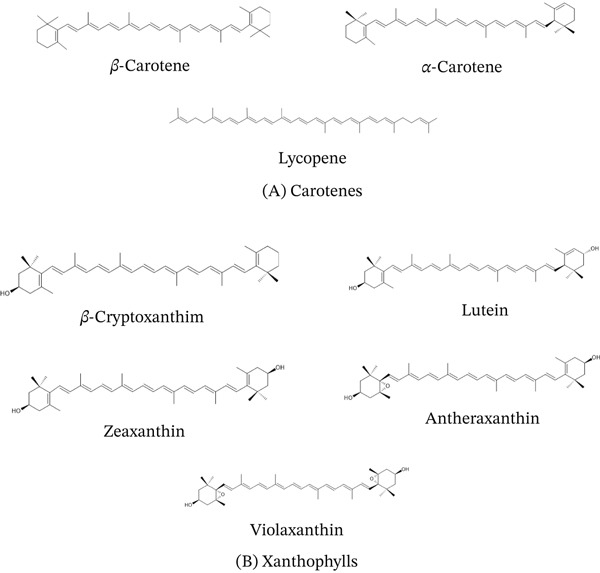
Chemical structure of carotenoids: (A) carotenes and (B) xanthophylls.

Carotenoids are isoprenoid molecules with several conjugated double bonds, which can have either a cyclic or acyclic structure. They are classified into two groups: carotenes, which are composed solely of carbon and hydrogen atoms, and xanthophylls, which are oxygenated derivatives of carotenes (Figure 4). These structural characteristics enable carotenoids to produce red, yellow, and orange pigmentation due to their high chemical reactivity and their ability to absorb light in the visible region of the electromagnetic spectrum [79].

Carotenoid biosynthesis in plants occurs in chloroplasts, although they are stored in chromoplasts (colored plastids), amyloplasts (starch storage plastids), and elaioplasts (lipid storage plastids) [80]. In plants, carotenoids play a role in stabilizing lipid membranes, forming lipoproteins, capturing light, and protecting photosystems from damage caused by reactive oxygen species [81].

Humans cannot synthesize carotenoids; as a consequence, they must be acquired by means of the consumption of fruits and vegetables. Carotenoids are vital in the human diet due to their vitamin A activity, antioxidant properties, and immunological benefits [82]. Vegetables can be classified based on their carotenoid content into four categories: low content (0–0.1 mg/100 g of fresh product), moderate content (0.1–0.5 mg/100 g), high content (0.5–2 mg/100 g), and very high content (> 2 mg/100 g) [83].

Lutein (8.9 mg/100 g; 73.41 mg/100 g), *β*‐carotene (12.9 mg/100 g; 3.72 mg/100 g), and zeaxanthin (1.7 mg/100 g) are the main carotenoids found in fresh basil leaves [84, 85]. A few studies have reported the presence of carotenoids in basil leaves. Abidoye et al. [86] found that adding 5 g of dried *O. basilicum* leaves to a roselle (*Hibiscus sabdariffa*) drink formulation produced a health‐promoting beverage enriched with bioactive compounds. The basil leaves also increased the total carotenoid content and antioxidant capacity of the drink.

## 4. Application of Basil in the Food Industry

### 4.1. Dried Leaves

Water is an essential component for the metabolic development of vegetables; however, high water content in food promotes rapid deterioration due to biological and chemical changes [87, 88]. To prevent such changes, it is necessary to reduce the amount of water molecules that are either bound or unbound to food constituents, thereby achieving low water activity (*a_w_*) levels [89, 90].

In this context, drying is an ancient food preservation method in which simultaneous heat and mass transfer between the drying air and the product promotes water removal [91]. During drying, water migrates from the interior to the surface of the product and evaporates into the surrounding environment. The increase in temperature causes water molecules to move and increases the partial vapor pressure of water in the product, leading to a reduction in water content and *a_w_* [92, 93]. The *a_w_* of a food predicts its microbial, enzymatic, and biochemical stability, supporting shelf life extension, improved storage stability, and more efficient production through volume reduction [90].

Drying operations are widely used in the food industry for vegetables, fruits, spices, and other products. However, process conditions—such as drying time, air temperature, air velocity, and relative humidity—must be adjusted to the intrinsic properties of each matrix to ensure the retention of flavor, aroma, and color, as well as the preservation of appearance and nutritional value [94, 95].

For aromatic plants, establishing ideal drying conditions is crucial, as these directly influence the quantity and quality of bioactive compounds. When carried out under optimal conditions, drying can contribute to a steady supply of the product and facilitate its commercialization [78, 94, 96].

Altay et al. [97] evaluated different drying methods for purple basil leaves, including sun drying, freeze‐drying, convection drying, and microwave drying. They found microwave drying to be the most suitable method, requiring the shortest time and lowest energy input. At 460 W, the process took 6 min, whereas at 600 W it required only 4.5 min, reaching a final moisture content of 3.09%. In contrast, sun drying was the least efficient, taking 10 h at 29°C–33°C and 24% relative humidity, and yielding a final moisture content of 8.01%.

Siti Mahirah et al. [98] investigated fresh basil leaves and those dried by freeze‐drying or in a vacuum oven. Using methanol as the solvent, they found that freeze‐drying best preserved the phenolic compounds, with a content of 54.46 mg of gallic acid/g, compared with 3.84 mg of gallic acid/g in fresh leaves and 38.26 mg of gallic acid/g in vacuum oven‐dried leaves. Antioxidant activity also varied with the drying method: freeze‐dried leaves showed the highest DPPH radical inhibition (92.60%) and the greatest ferric reduction power (1160.95 *μ*g FeII/g).

Therefore, the drying method plays a critical role in the degradation of bioactive compounds. Sharma et al. [77] examined the effects of sun drying (12.2°C–21°C, 37%–98% relative humidity), oven drying (45°C, 50°C, and 55°C), and microwave drying on ascorbic acid, they also examined the effects of total phenolic compounds, and chlorophylls contents in purple basil. They reported that microwave drying preserved the highest levels of chlorophyll (31.8 mg/g) and total phenolic compounds (174.91 mg/100 g) while requiring the shortest drying time. In contrast, natural sun drying retained the highest ascorbic acid content (207.45 mg/100 g).

### 4.2. Essential Oil

Essential oils are complex liquid compounds formed by secondary metabolites in plants, biosynthesized in various plant organs, such as buds, flowers, leaves, stems, fruits, seeds, branches, bark, and stored in secretory cells, cavities, channels, epidermal cells, or glandular trichomes [99, 100]. They are primarily composed of terpenes and terpenoids, along with oxygenated derivatives such as alcohols, aldehydes, esters, ketones, phenols, and oxides. These compounds impart biological properties, as well as volatile characteristics that provide a distinctive odor and flavor [99, 101].

Various extraction techniques have been employed to obtain essential oils, including hydrodistillation, cold pressing, solvent extraction, microwave‐assisted extraction, ultrasound‐assisted extraction, and supercritical fluid extraction [102]. The choice of extraction method is crucial, as factors such as temperature, oxygen, light, and pH can alter the structure of essential oils, potentially leading to discoloration, changes in flavor and odor, loss of bioactive compounds, and physical modifications such as increased viscosity [102, 103]. Hydrodistillation is a traditional and straightforward method widely used for essential oil extraction. This process is conducted in a Clevenger apparatus, which allows distillation by circulating water vapor, facilitating the quantitative recovery of volatile compounds [104].

The bioactive compounds in essential oils exhibit diverse biological activities, including antimicrobial, antioxidant, anti‐inflammatory, cancer chemoprotective, cytotoxic, allelopathic, repellent, and insecticidal effects [105–107]. Consequently, essential oils find applications in human health, green chemistry, and sustainable agriculture. Adetuyi et al. [108] reported the use of essential oils extracted from culinary herbs as biopreservatives in food.

The essential oil of basil leaves contains volatile compounds such as methylchavicol and geranial, which exhibit antioxidant activity and aid in scavenging free radicals [109]. Koroch et al. [110] identified methylchavicol and eugenol as the major constituents of *O. basilicum* leaves, whereas Perveen et al. [111] reported methylchavicol and linalool. Lawrence et al. [112] identified 32 constituents, with terpinen‐4‐ol, eucalyptol, and *α*‐terpineol as predominant. Shahrajabian et al. [6] highlighted the variability in the chemical composition of basil leaves, attributing it to factors such as cultivar, cultivation method, geographic origin, and harvest time.

Kanmaz et al. [113] investigated the chemical composition of purple basil essential oil and its antioxidant and antidiabetic activities. Antioxidant activity was confirmed by ABTS and DPPH assays, with values of 51.12 and 210.01 mg TE/g, respectively. Antidiabetic activity was assessed in streptozotocin‐induced diabetic rats, with a control group for comparison. Administration of purple basil essential oil (70 mg/kg) inhibited *α*‐glucosidase by 79.61% and *α*‐amylase by 62.64% while also providing protective effects for pancreatic cells. Blood sugar levels decreased from 370.2 mg/dL at baseline to 272.8 mg/dL after 28 days, representing a 26.3% reduction.

### 4.3. Infusion

Foods and beverages with health‐promoting properties have attracted increasing attention. Tea, the second most consumed nonalcoholic beverage worldwide, is produced from the shoots and leaves of *Camellia sinensis*. It can be classified according to the degree of fermentation and is valued for both dietary and medicinal applications [114, 115]. In addition, aromatic plants from the Lauraceae, Umbelliferae, Lamiaceae, Myrtaceae, and Compositae families have also been explored for tea production because of their functional properties [116].

Herbal teas or infusions are prepared by steeping dried parts of aromatic plants—such as roots, leaves, flowers, and fruits—in hot or boiling water [117]. However, the preparation and consumption of these infusions can vary according to cultural traditions and regional preferences [102].

Several plant species are commonly used in the preparation of infusions, including mint (*Mentha spicata* L.), chamomile (*Matricaria chamomilla* L.), lemon balm (*M. officinalis* L.), rosemary (*Rosmarinus officinalis* L.), cinnamon (*Cinnamomum zeylanicum*), and ginger (*Zingiber officinale*) [102, 116]. However, studies on infusions made with purple basil (*O. basilicum*) are limited. Costa et al. [118] evaluated the terpene profile of *O. basilicum* tea under different preparation (infusion time) and storage conditions (refrigerated and frozen) and found that the composition of terpenes, including limonene and terpinene, remained stable in the infusions.

Gulhan et al. [29] evaluated the total phenolic content, total monomeric anthocyanins, and instrumental color of infusions prepared from dried purple basil leaves. They recommended using leaves dried by microwave or shade methods, with an optimal infusion time of 45 min in hot water, to maximize bioactive compound levels. Infusion from microwave‐dried leaves contained 308.35 mg/L of total phenolics and 19.69 mg/L of monomeric anthocyanins, whereas those shade‐dried leaves yielded 324.76 and 22.26 mg/L, respectively.

### 4.4. Minimum Processing

Minimal processing of vegetables is an alternative for convenient consumption, preferred by consumers with limited time who seek a healthier diet rich in vitamins, fiber, minerals, and antioxidants [119–121]. This processing method is a technological approach that offers practical and safe food products while meeting consumer expectations regarding quality and convenience [121, 122].

The preparation of minimally processed vegetables involves removing inedible parts, with key processing steps including selection, prewashing, cutting or peeling, cleaning, rinsing, centrifugation, packaging, and refrigeration [123]. However, the cutting process during preparation can induce mechanical stress, triggering physiological and biochemical changes, microbial deterioration, and modifications in sensory attributes such as color, texture, odor, and flavor—factors that influence consumer preference [119, 124, 125].

Various preservation methods, including physical and chemical treatments, can be applied to maintain sensory characteristics and extend the shelf life of vegetables. These include packaging techniques that create a modified atmosphere by regulating the permeability to O_2_, CO_2_, ethylene, and water vapor, as well as vacuum sealing; refrigeration (0°C–10°C), which slows enzymatic browning and microbial growth; and the use of chemical agents with antioxidant, acidifying, chelating, or enzyme‐inhibiting properties [126–128].

Temperature plays a crucial role in ensuring the preservation of minimally processed vegetables. Low temperatures inhibit spoilage agents by reducing the rate of oxidative, enzymatic, and microbiological reactions, although these processes are not entirely halted [129, 130].

Both oxygen exposure and water content in food accelerate metabolic degradation. In this context, packaging acts as a physical barrier against oxygen and other external factors, helping to maintain product quality while improving handling convenience. Studies have examined the effects of modified atmosphere packaging on minimally processed vegetables such as lettuce (*Lactuca sativa* L.) [131], cabbage (*Brassica oleracea* var. *capitata*) [132], eggplant (*Solanum melongena*) [126], and green beans (*Phaseolus vulgaris* L.) [133].

Chemical treatments for food preservation involve the addition of substances that maintain or enhance the chemical, physical, and biological characteristics of the product [134]. Food additives can be classified based on their function as antioxidants, antimicrobials, acidulants, thickeners, humectants, antihumectants, colorants, flavorings, sweeteners, and stabilizers [135].

Antioxidants are an effective preservation strategy, as they reduce respiration rates, inhibit enzymatic activity, limit nutrient and pigment losses, and may also provide antimicrobial effects. This approach typically involves immersing vegetables in solutions containing organic acids, minerals, or their combinations [136].

Weak organic acids are regarded as safe for human consumption, as they partially dissociate in aqueous solutions. They function as acidifying and antioxidant agents, with efficiency depending on their concentration and chemical composition. The most commonly used organic acids in vegetable preservation include ascorbic, citric, salicylic, and oxalic acids [136, 137].

Several studies have demonstrated the efficacy of organic acids in preserving vegetables. Souza et al. [138] and Souza et al. [139] investigated the effects of ascorbic acid combined with refrigeration on minimally processed kale and green onions. Anuar et al. [140] used a combination of ascorbic acid, citric acid, EDTA, and benzoic acid in controlled proportions to prevent browning in ginger flower puree (*Etlingera elatior*). Abdel‐Hamid [141] evaluated the quality of mint and sage treated with citric acid, salicylic acid, and chitosan, whereas Uscanga‐Soso et al. [142] demonstrated that ascorbic acid treatments with pH control extended the shelf life of minimally processed eggplants.

Research on the minimal processing of basil is limited. The only study identified was a review by Brindisi and Simon [143], which discusses strategies to extend the shelf life and quality of purple basil, including storage conditions (temperature, lighting, controlled atmosphere, and packaging), heat treatments, phytohormone application, 1‐methylcyclopropene treatment, nanotechnology, and production methods.

### 4.5. Use of Basil as an Ingredient in Food Products

Basil (*O. basilicum*) is a plant species with significant potential for application in the food industry. After harvesting, its leaves can be used fresh, sold in a minimally processed form, dried, or utilized for the preparation of infusions, seasonings, and essential oil extraction. This species has been studied due to its diverse functional properties, as outlined in Table 2.

**Table 2:** Applications of basil (*O. basilicum*) in the food industry.

Application form	Product	Functionality	Reference
Dry leaves	Drink	Antioxidant and antidiabetic activity	Demircan et al. [144]
Microencapsulated extract	Cream cheese	Antioxidant and microbial activity	Popescu et al. [21]
Fresh and dried leaves	Roselle (*Hibiscus sabdariffa*) drink	Increase carotenoid content and antioxidant activity	Abidoye et al. [86]
Ethanolic extract of leaves	Biodegradable film based on bovine gelatin	Antioxidant and microbial activity	Ebrahimi et al. [19]
Microencapsulated essential oil	Ice cream	Antioxidant activity	Veena et al. [145]
Essential oil	Ground beef	Antioxidant activity	Falowo et al. [146]
Dry leaves	Buffalo milk cheese	Antioxidant activity and pH control	Ribas et al. [147]
Infusion	Kombucha beverage	Antioxidant activity	Yıkmış and Tuğgüm [148]
Dried leaves and infusion	Yogurt	Antioxidant activity and aid in the fermentation process	Gurkan et al. [149]
Essential oil	Chicken meat	Antimicrobial activity	Stojanovic‐Radic et al. [150]
Essential oil	Biodegradable film based on chitosan	Antimicrobial activity and antioxidant activity	Hemalatha et al. [151]

Basil represents a valuable raw material for the food industry due to its health‐promoting properties. Demircan et al. [144] developed a sugar‐free beverage from dried basil leaves, resulting in a product rich in bioactive compounds with biological properties. The quantified levels of total phenolic compounds, total carotenoids, vitamin C, and total chlorophylls were 292.17 mg GAE/g, 2303.16 mg/100 g, 1860 mg/100 mL, and 39.79 mg/100 g, respectively. In vitro analyses revealed a free radical scavenging activity of 0.156 mg/mL (IC_50_) and an antidiabetic activity of 1.67 mg/mL (IC_50_). Additionally, the beverage was accepted by tasters regarding color, texture, flavor, aroma, and overall acceptability.

Popescu et al. [21] applied microencapsulated basil extract to cheese to improve preservation and confer functional properties. The extract contained phenolic compounds such as methyl‐rosmarinate, rosmarinic acid, rosemary, carnosol, dehydrodiferulic acid, and chicoric acid. It showed antioxidant activity of 644.75 mM Trolox/g (DPPH) and 8.95 mM Trolox/g (ABTS). Incorporation into cheese enhanced its antioxidant activity, inhibited microorganisms including *Staphylococcus aureus*, *Bacillus cereus*, *Enterococcus faecalis*, and *Escherichia coli*, delayed degradation, and improved sensory characteristics.

Abidoye et al. [86] evaluated the antioxidant activity of *O. basilicum* leaves by incorporating dried basil at 5%, 10%, and 15% into a roselle (*H. sabdariffa*) beverage, alongside a control without basil. The basil‐enriched formulations showed higher total carotenoid levels than the control (5.06 mg/100 g), with values of 7.86 mg/100 g (5% basil), 10.56 mg/100 g (10%), and 16.12 mg/100 g (15%). The total phenolic content and antioxidant activity followed the same trend, increasing from 21.45 mg/g catechin equivalent (CE) in the control to 29.46 mg/g CE (5% basil), 38.21 mg/g CE (10%), and 46.64 mg/g CE (15%).

For the beverage, antioxidant activity assessed by the ABTS radical scavenging method ranged from 25.26 *μ*mol TEC/g in the roselle‐only drink to 86.44 *μ*mol TEC/g in the drink with 15% basil leaves. Similar results were obtained with the ORAC method, which showed values of 18.46 *μ*mol TEC/g for the control and 56.15 *μ*mol TEC/g for the 15% basil formulation. Abidoye et al. [86] concluded that the roselle‐basil beverage is a healthy product with strong free radical scavenging potential, high levels of total carotenoids and phenolic compounds, and that the formulation with 5% basil leaves offered the best sensory acceptability.

Due to their chemical composition, purple basil leaves can be incorporated into biodegradable films to enhance food preservation and indicate product deterioration. Ebrahimi et al. [19] developed biodegradable bovine gelatin films containing purple basil leaf extracts to monitor chicken fillet freshness. The films initially exhibited a purple coloration with 74.71 mg/L of anthocyanins, and analysis of release rates showed that 25.58%–26.22% of anthocyanins were released within 200 min.

Therefore, Ebrahimi et al. [19] developed a pH‐sensitive film that changed color from purple to green as pH increased during storage. Applied to meat products, this color transition signaled spoilage because protein degradation produces biogenic amines that raise the pH. The TVB‐N content, a spoilage indicator, increased from 8.70 to 37.81 mg N/100 after 16 h at room temperature, corresponding to the initial purple and final green colors. Moreover, incorporation of purple basil extract enhanced the films′ antioxidant and antibacterial properties.

Veena et al. [145] investigated basil essential oil as a functional ingredient in ice cream to enhance antioxidant properties. Three formulations were tested: a control without basil oil, one with 0.3% (v/v) basil oil, and one with 0.5% (w/v) spray‐dried microencapsulated basil oil. The 0.5% microencapsulated formulation showed the highest antioxidant activity, with 94.57% DPPH radical inhibition and a total phenolic content of about 75 *μ*g GAE/mL. Sensory evaluation revealed high acceptance scores for color, appearance, texture, flavor, mouthfeel, and overall acceptability (all above 7.0). The authors concluded that ice cream containing microencapsulated essential oil is both nutritionally beneficial and sensory well‐accepted.

Falowo et al. [146] evaluated the effect of *O. basilicum* essential oil on color stability and lipid oxidation in ground meat during storage. Four treatments were prepared: a control (no oil) and samples with 2%, 4%, and 6% essential oil, stored in polyethylene bags at 4°C and analyzed on Days 0, 2, 4, and 7. The oil was rich in bioactive compounds, including estragole (41.40%), 1,6‐octadiene‐3‐ol, 3‐7‐dimethyl (29.49%), and trans‐*α*‐bergamot (5.32%), with known antioxidant and antimicrobial properties. Results showed that essential oil enhanced color stability, as b* and L* values remained higher than the control throughout storage, and reduced lipid oxidation even after 7 days.

Ribas et al. [147] incorporated powdered dried basil leaves into buffalo milk cheese and observed increases in antioxidant activity and total phenolic content. On Day 1, phenolic levels were 0.39 mg GAE/g in the control and 0.61 mg GAE/g in the sample with 7.5 g basil. After 21 days, the control decreased to 0.23 mg GAE/g, whereas the basil sample maintained a higher level of 0.38 mg GAE/g. Antioxidant capacity, assessed by the ABTS method, was 12.03% for the control and 53.03% for the basil sample on Day 1, and 8.53% and 15.80%, respectively, after 21 days. Basil addition also improved pH, color, texture, and microstructure of the cheese.

Purple basil infusion has also been incorporated into kombucha beverages. Yıkmış and Tuğgüm [148] added basil infusion after fermentation to enhance bioactive compound and antioxidant activity of kombucha. On Day 1 of storage, kombucha with 100% purple basil infusion showed the highest levels of total phenolics (266.21 mg GAE/L), total flavonoids (31.01 mg CE/L), and DPPH inhibition (68.09%). After 30 days at 4°C, only slight reductions were observed, with values of 260.11 mg GAE/L, 29.70 mg CE/L, and 64.19%, respectively, confirming the infusion′s potential to improve product quality.

Gurkan et al. [149] incorporated purple basil powder and aqueous extract into yogurt formulations and evaluated physicochemical, rheological, antioxidant, color, and phenolic properties during 21 days of refrigerated storage at 4°C. Physicochemical and rheological properties showed no significant changes. However, yogurts with 1% powdered basil leaves had the highest levels of total phenolic (3.10 mg GAE/g), total flavonoids (0.36 mg CE/10 g), and antioxidant activity (2.94 mmol TE/kg) at the beginning of storage, compared with formulations containing 0.4% or 1% aqueous extract, 0.4% powder, and the control. After 21 days, the 1% powdered basil yogurt maintained its chemical properties, confirming that purple basil enhanced the antioxidant properties of yogurt.

Stojanovic‐Radic et al. [150] reported that incorporating basil essential oil into raw chicken meat inhibited *Salmonella enteritidis* after 24 h of storage at 4°C and stabilized lipid oxidation, reducing cooking losses. Similarly, Hemalatha et al. [151] developed a biodegradable chitosan film containing purple basil essential oil for active food packaging. At 0.1% concentration, the oil inhibited fungal growth, whereas 0.5% incorporation enhanced antioxidant activity, achieving about 43% inhibition in the DPPH assay. The addition of purple basil essential oil also improved the film′s thermal and mechanical properties.

The use of *O. basilicum* in foods applications is supported by its long history of culinary consumption and by the safety evaluation of several of its volatile constituents as flavoring substances within internationally recognized regulatory frameworks [152]. In the United States, compounds commonly present in basil essential oil, such as linalool and eugenol, have been evaluated under the generally recognized as safe (GRAS) framework for their intended uses in food, subject to established conditions of use and current good manufacturing practices [153]. Similarly, the European Food Safety Authority (EFSA) recommends that the safety assessment of botanical preparations intended for food applications be based on comprehensive chemical characterization, intended conditions of use, and estimated dietary exposure to ensure consumer protection [154].

In Brazil, although basil is widely consumed as a culinary herb, concentrated extracts or ingredients intended for use in foods with functional or health‐related claims are subject to the regulatory requirements established by ANVISA (National Health Surveillance Agency), including applicable safety assessment procedures [155]. Despite the widespread use of basil as a food ingredient, safety considerations remain particularly important for concentrated extracts and essential oils. Certain basil chemotypes contain relatively high concentrations of estragole (methyl chavicol), a naturally occurring phenylpropanoid that has been the subject of toxicological concern because of evidence of genotoxic and carcinogenic effects observed primarily in experimental animal studies conducted at high exposure levels [156].

Nevertheless, the levels of estragole typically associated with conventional culinary consumption of basil are generally much lower than those used in toxicological studies, and the available evidence indicates that the overall safety of basil consumption is supported by its long history of dietary use and previous toxicological evaluations [16, 152]. Accordingly, the development of functional foods, active packaging systems, and other innovative applications incorporating purple basil should emphasize standardized extraction procedures, compositional characterization, exposure and dose assessment, and compliance with applicable regulatory requirements to ensure both product efficacy and consumer safety [153–156].

## 5. Conclusion and Future Perspectives

The literature identifies purple basil (*O. basilicum*) as a rich source of bioactive compounds, particularly phenolics, which underpin its strong antioxidant and antimicrobial properties. Its chemical composition varies with geographical origin and cultivar, influencing its functional potential. Purple basil can be applied in the food industry in fresh or dried forms, as infusions, or as a source of essential oil, and it can also be marketed as a minimally processed product. However, further research is needed to optimize preservation strategies and extend shelf life. Studies have demonstrated the successful incorporation of basil leaves, extracts, infusions, and essential oils into diverse food products—including ice cream, cheese, yogurt, and meat products—as well as into biodegradable films, where they enhance antioxidant and antimicrobial properties. Collectively, these findings highlight purple basil as a promising raw material for food applications.

However, the scientific evidence specifically addressing purple basil cultivars remains less extensive than that available for *O. basilicum* as a whole. Therefore, the broader literature on *O. basilicum* was used where appropriate to provide mechanistic and technological context. Nonetheless, further investigation is needed to elucidate the biological activities of purple basil, optimize formulations containing *O. basilicum* derivatives, and support its development as a functional ingredient.

## Author Contributions

Rosane Patricia Ferreira Chaves researched and analyzed the selected scientific publications and wrote the manuscript. Elivaldo Nunes Modesto Junior, Gustavo Araujo Pereira, and Rosinelson da Silva Pena analyzed the scientific publications used to produce the manuscript and provided critical feedback.

## Funding

This work was supported by the Coordenação de Aperfeiçoamento de Pessoal de Nível Superior through a scholarship awarded to Rosane P. F. Chaves (88887.605381/2021‐00).

## Disclosure

All authors reviewed the manuscript.

## Consent

The authors have nothing to report.

## Conflicts of Interest

The authors declare no conflicts of interest.

## Data Availability

Data sharing is not applicable to this article as no datasets were generated or analyzed during the current study.
